# Clinical findings, viral load, and outcomes of COVID-19: Comparison of patients with negative and positive initial chest computed tomography

**DOI:** 10.1371/journal.pone.0264711

**Published:** 2022-03-03

**Authors:** Cherry Kim, Ji-Yeon Kim, Eun Joo Lee, Yu Min Kang, Kyoung-Ho Song, Eu Suk Kim, Eun Jin Kim, Seungsoo Sheen, Yoo Ra Lee, BeoDeul Kang, Joon Ho Kim, Myoung Lyeol Woo, Chul Hee Park, Soohoon Kwon, Eun Ju Choo, Tark Kim, Donghoon Kim, Hong Sang Oh, Won Suk Choi

**Affiliations:** 1 Department of Radiology, Ansan Hospital, Korea University College of Medicine, Gyeonggi-do, South Korea; 2 Division of Infectious Diseases, Department of Internal Medicine, Seongnam Citizens Medical Center, Seongnam-si, Gyeonggi, South Korea; 3 Department of Pediatrics, Seongnam Citizens Medical Center, Seongnam-si, Gyeonggi-do, South Korea; 4 Division of Infectious Diseases, Department of Internal Medicine, Myongji hospital, Goyang-si, Gyeonggi-do, South Korea; 5 Department of Internal Medicine, Seoul National University Bundang Hospital, Seoul National University College of Medicine, Seongnam-si Gyeonggi-do, South Korea; 6 Division of Infectious Diseases, Department of Internal Medicine, Ajou University School of Medicine, Suwon-si, Gyeonggi-do, South Korea; 7 Department of Pulmonary and Critical Care Medicine, Ajou University School of Medicine, Suwon-si, Gyeonggi-do, South Korea; 8 Department of Internal Medicine, Gyeonggi Provincial Medical Center, Ansung Hospital, Anseong-si, Gyeonggi-do, South Korea; 9 Department of Surgery, Gyeonggi Provincial Medical Center Uijeongbu Hospital, Uijeongbu-si, Gyeonggi-do, South Korea; 10 Department of Internal Medicine, Gyeonggi Provincial Medical Center Uijeongbu Hospital, Uijeongbu-si, Gyeonggi-do, South Korea; 11 Department of Internal Medicine, Icheon Medical Center, Icheon-si, Gyeonggi-do, South Korea; 12 Department of Internal Medicine, Soonchunhyang University Bucheon Hospital, Bucheon-si, South Korea; 13 Department of Internal Medicine, Armed Forces Capital Hospital, Seongnam-si, Gyeonggi-do, South Korea; 14 Division of Infectious Diseases, Department of Internal Medicine, Korea University College of Medicine, Korea University Ansan Hospital, Ansan-si, Republic of Korea; Al-Azhar University, EGYPT

## Abstract

Reports detailing the clinical characteristics, viral load, and outcomes of patients with normal initial chest CT findings are lacking. We sought to compare the differences in clinical findings, viral loads, and outcomes between patients with confirmed COVID-19 who initially tested negative on chest CT (CT negative) with patients who tested initially positive on chest CT (CT positive). The clinical data, viral loads, and outcomes of initial CT-positive and CT-negative patients examined between January 2020 and April 2020 were retrospectively compared. The efficacy of viral load (cyclic threshold value [Ct value]) in predicting pneumonia was evaluated using receiver operating characteristic (ROC) curve and area under the curve (AUC). In total, 128 patients underwent initial chest CT (mean age, 54.3 *±* 19.0 years, 50% male). Of those, 36 were initially CT negative, and 92 were CT positive. The CT-positive patients were significantly older (*P* < .001) than the CT-negative patients. Only age was significantly associated with the initial presence of pneumonia (odds ratio, 1.060; confidence interval (CI), 1.020-1-102; *P* = .003). In addition, age (OR, 1.062; CI, 1.014–1.112; *P* = .011), fever at diagnosis (OR, 6.689; CI, 1.715–26.096; *P* = .006), and CRP level (OR, 1.393; CI, 1.150–1.687; *P* = .001) were significantly associated with the need for O_2_ therapy. Viral load was significantly higher in the CT-positive group than in the CT-negative group (*P* = .017). The cutoff Ct value for predicting the presence of pneumonia was 27.71. Outcomes including the mean hospital stay, intensive care unit admission, and O_2_ therapy were significantly worse in the CT-positive group than in the CT-negative group (all *P* < .05). In conclusion, initially CT-negative patients showed better outcomes than initially CT-positive patients. Age was significantly associated with the initial presence of pneumonia, and viral load may help in predicting the initial presence of pneumonia.

## Introduction

Several reports have shown that chest CT yielded positive findings suggesting COVID-19 pneumonia even in patients with false-negative real-time reverse-transcriptase polymerase-chain reaction (rRT-PCR) results [1–4]. In contrast, several studies have shown that rRT-PCR-confirmed COVID-19 may be associated with normal chest CT findings in early-stage infection. Ai et al. reported that 3% (21 of 601) of patients with positive rRT-PCR results had normal chest CT findings [5]. Furthermore, in the study by Bernheim et al. [6], 56% (20 of 36) of patients with confirmed COVID-19 showed normal CT findings 0–2 days after symptom onset. Fang et al. also reported normal chest CT findings 3 days after symptom onset in 2% (1 of 51) of patients [7]. In addition, a recent study reported that 25% of patients with asymptomatic COVID-19 had no abnormal findings on chest CT [8].

However, only few reports have been published on the follow-up findings or prognosis of patients with normal initial chest CT findings. Pan et al. reported that lung abnormalities developed approximately 4 days after the first chest CT scan with normal findings in 4 patients (19%, 4 of 21) [9]. Yang et al. reported that of 17 patients (11.4%, 17 of 149) with normal chest CT findings on admission, 5 patients developed pneumonia over an average of 7 days, whereas 12 patients remained CT negative even after 10 days [10]. If the viral load and outcome are worse in patients who are positive on initial CT than in patients who are negative on initial CT, those who are positive on initial CT may be managed with slightly more caution. In addition, if factors related to positivity on initial CT are identified, patients with these factors can also be cared for with special attention. To our knowledge, reports detailing the clinical characteristics, viral load, and outcomes of patients with normal initial chest CT findings compared with those of patients with positive initial CT findings are lacking.

Therefore, we aimed to compare the differences in clinical findings, viral load, and outcomes between patients with confirmed COVID-19 with normal initial chest CT findings with those with positive initial CT findings initially.

## Materials and methods

This retrospective study was approved by the Institutional Review Board of Korea University Ansan Hospital (KUMC), and informed consent for the use of clinical data was waived (approval number: 2020AS0108).

### Study population

In total, 490 patients with rRT-PCR-confirmed COVID-19 were admitted to 9 hospitals in Gyeonggi Province in Korea between January 2020 and April 2020. During this period, according to the policy of the Korean government, all patients with confirmed COVID-19 were admitted to the hospital regardless of their symptoms. Among these patients, chest CT was performed in 128 patients (mean days from initial diagnosis to chest CT, 2.99 ± 3.26 days). Follow-up chest CT scans and/or chest radiographs (CXRs) were performed at least once per week until discharge for all patients. In most cases, if there was an abnormality on CXRs or if respiratory symptoms were present without any abnormalities on CXRs, CT was performed to confirm the presence of pneumonia; this decision was made by the clinicians from each hospital.

The clinical and epidemiologic characteristics of all the patients, such as age, sex, underlying disease (diabetes mellitus, heart failure, hypertension, chronic heart disease [without heart failure], asthma, chronic obstructive pulmonary disease, malignancy, and dementia), symptoms at the time of diagnosis and during follow up, and laboratory examination findings including the white blood cell (WBC) count, presence of leukocytosis (WBC count >10.0 × 10^3^/μL), leukopenia (WBC count < 4.0×10^3^/μL), or lymphopenia (absolute lymphocyte count < 1000/mm^3^), and C-reactive protein (CRP) level at diagnosis of all the included patients were recorded.

**Fig 1:**
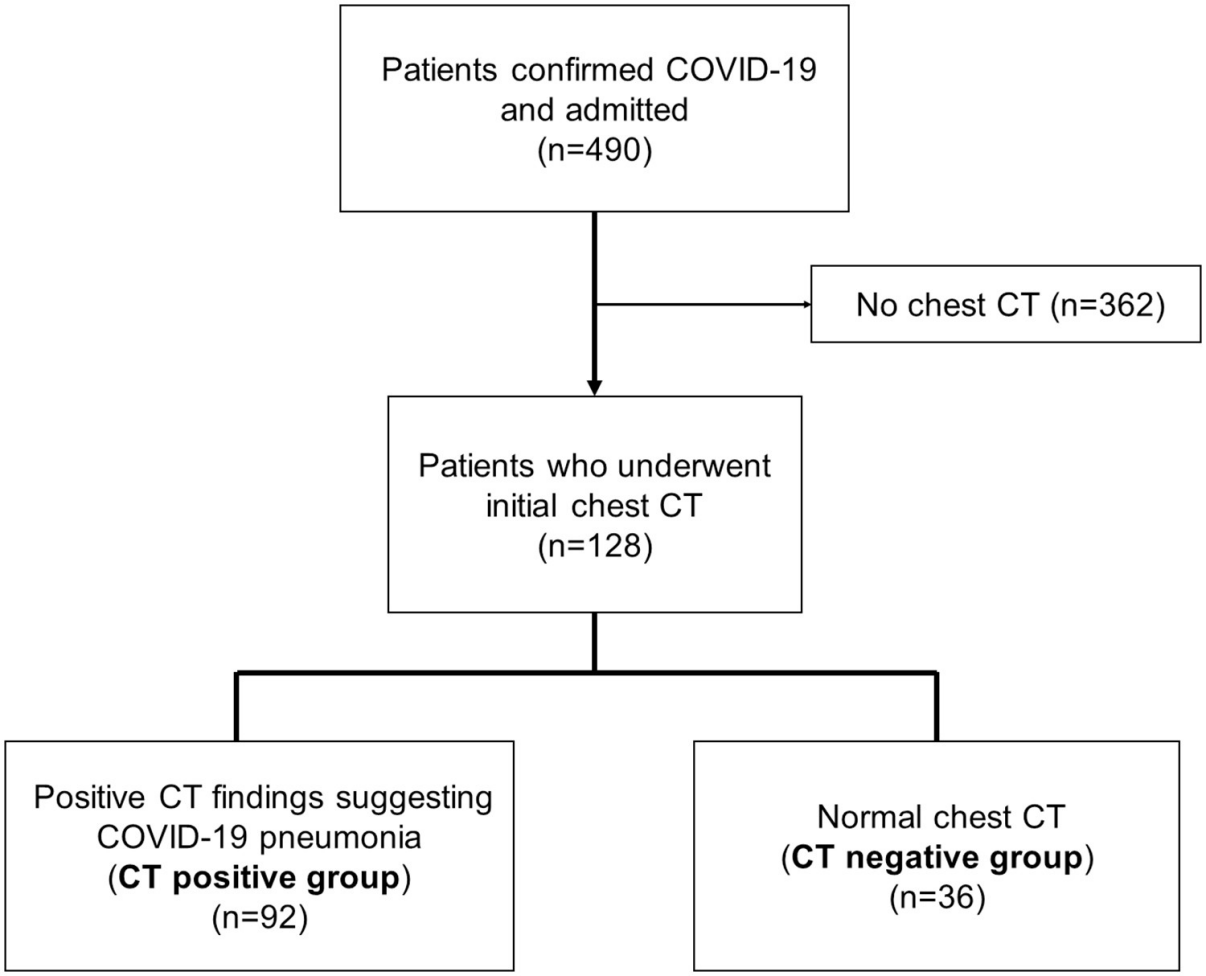
Study population accrual process. This figure summarizes the accrual process of the study population.

Fig 1 summarizes the accrual process of the study population.

### Image interpretation

The radiology reports of all the initial and follow-up chest CT scans and CXRs used in real clinical practice were evaluated by a board-certified thoracic radiologist (C.K.). For opacity pattern analysis, all lung parenchymal lesions were categorized as follows: no lesion, only ground-glass opacity (GGO), both GGO and consolidation, and only consolidation. The number of affected lobes (0–5), > 2 lobes affected, bilateral lung disease, opacification distribution (rounded morphology, linear opacities, crazy-paving pattern, peripheral or peribronchovascular distribution, and cryptogenic organizing pneumonia pattern), and other findings (pleural effusion, lymphadenopathy, pulmonary emphysema, and pulmonary fibrosis) were recorded.

Patients were identified as CT negative when no lesion was identified in the lung parenchyma, suggesting the absence of COVID-19 infection. All other aforementioned lung parenchymal categories were considered CT positive because they suggest COVID-19 pneumonia. Among 128 patients who underwent initial chest CT, 92 patients had positive CT findings indicative of COVID-19 pneumonia (CT-positive group), whereas 36 patients had normal chest CT findings (CT-negative group).

### CT protocol

All CT examinations were performed using a multidetector CT scanner with 64 or more channels (SOMATOM Definition edge, SOMATOM Definition AS, SOMATOM Definition AS+, SOMATOM Scope Power, or SOMATOM Force [Siemens Medical Solutions, Erlangen, Germany], Aquilion ONE [Canon medical system, Otawara, Tochigi, Japan], Brilliance CT 64, or Ingenuity Core [Philips Healthcare, Cleveland, Ohio, USA]) with a tube potential of 120 kVp and tube current of 30–200 mAs. All images were obtained in a caudo-cranial direction from the lung base through the thoracic inlet level during a single inspiratory breath-hold.

### Collection and analysis of respiratory specimens

For all patients, respiratory specimens were collected from the upper (nasopharyngeal/oropharyngeal swab) and lower respiratory tracts (sputum) at the time of diagnosis. Using these specimens, rRT-PCR analysis was performed to detect SARS-CoV-2 using the PowerChek 2019-nCoV Real-time PCR kit (Kogenebiotech, Seoul, Korea), DiaPlexQ 2019-nCoV Assay (EDGC, Seoul, South Korea) or Allplex 2019-nCoV Assay (Seegene, Seoul, South Korea). Medical laboratory experts interpreted the results as negative, positive, or inconclusive. All patients included in this study were tested several times, and rRT-PCR was periodically performed to filter out false positives.

To assess viral load, the cycle threshold value (Ct value) was identified. The Ct value is defined as the number of cycles required for the fluorescence signal to cross the threshold, indicating viral load. The viral load in specimens from the upper and lower respiratory tracts was estimated using the Ct value of the N gene, with lower Ct values indicating a higher viral load [11].

### Clinical outcomes

The mean number of follow-up days for the entire study population was 30.3 ± 16.8 days. Several indicators, such as discharge, intensive care unit (ICU) admission, death, and whether O_2_ therapy, such as nasal prongs, facial mask, high flow nasal prongs, mechanical ventilator (MV), and extracorporeal membrane oxygenation (ECMO), was administered during admission, were analyzed during these follow-up periods to assess outcomes. All patients were discharged from the admitted hospital or transferred to a nursing hospital only after 2 consecutive negative rRT-PCR tests conducted at 24-hour or longer intervals; both outcomes were considered “discharged” in this study. Severity classification was performed according to World Health Organization (WHO) guidelines [12]. "Critical COVID-19" is defined by the criteria for acute respiratory distress syndrome (ARDS), sepsis, septic shock, or other conditions that would normally require the provision of life-sustaining therapies such as MV or vasopressor therapy. "Severe COVID-19" is defined by any of O_2_ saturation < 90% on room air, respiratory rate > 30 breaths/min, or signs of severe respiratory distress. "Non-severe COVID-19" is defined as the absence of any criteria for severe or critical COVID-19.

### Statistical analysis

To compare the clinical and epidemiologic characteristics, laboratory findings, viral loads, outcomes, and CT findings between the CT-positive and CT-negative groups, Student’s t-test or the Mann-Whitney U test were used for continuous variables, and the chi-square test or Fisher’s exact test were used for categorical variables. The diagnostic performance of the lowest Ct values for predicting COVID-19 pneumonia was analyzed using receiver operating characteristic (ROC) curves and the area under the curve (AUC). Factors associated with positivity on initial CT and the need for O_2_ therapy were identified by univariate and multivariate logistic regression analyses for all patients. A *P* value < .05 was considered significant. All statistical analyses were performed with SPSS software (version 20.0; SPSS, Chicago, IL) and MedCalc (version 19.1.3; MedCalc Software Ltd, Acacialaan, Belgium).

## Results

### Clinical characteristics and laboratory findings

The clinical findings, epidemiologic characteristics, and laboratory findings of both groups are summarized in Table 1. The mean age of the CT-positive group was significantly older than that of the CT-negative group (58.8 ± 15.7 vs. 43.0 ± 22.2 years; *P* < .001). The proportion of patients with comorbidities was higher in the CT-positive group than in the CT-negative group (58.3% vs. 29.3%; *P* < .002). A significantly higher proportion of patients had hypertension in the CT-positive group than in the CT-negative group (44.6% vs. 22.2%; *P* = .026).

**Table 1:** Clinical findings, epidemiologic characteristics, and laboratory findings of the study population.

	CT negative	CT positive	*P* value
Age (years)	43.0 *±* 22.2	58.8 *±* 15.7	< .001
Male sex	19 (52.8%)	45 (48.9%)	.844
*Underlying disease*			
None	21 (58.3%)	27 (29.3%)	< .002
DM	6 (16.7%)	17 (18.5%)	>.999
Heart failure	1 (2.8%)	0	.281
HTN	8 (22.2%)	41 (44.6%)	.026
Chronic heart disease	0	6 (6.5%)	.184
Asthma	0	3 (3.3%)	.559
COPD	1 (2.8%)	2 (2.2%)	>.999
Malignancy	1 (2.8%)	7 (7.6%)	.440
Dementia	3 (8.3%)	6 (6.5%)	.710
*At the time of diagnosis*			
None	3 (8.3%)	12 (13.2%)	.756
Fever	7 (19.4%)	36 (39.6%)	.038
Cough	15 (41.7%)	31 (34.1%)	.422
Sputum	8 (22.2%)	19 (20.9%)	>.999
Dyspnea	0	12 (13.2%)	.019
Sore throat	4 (11.1%)	14 (15.4%)	.778
Rhinorrhea	4 (11.1%)	5 (5.5%)	.271
Chest pain	1 (2.8%)	4 (4.4%)	>.999
Myalgia	6 (16.7%)	23 (25.3%)	.298
Fatigue	3 (8.3%)	9 (9.9%)	.787
Nausea/vomiting	2 (5.6%)	3 (3.3%)	.555
Abdominal pain	0	1 (1.1%)	.528
Diarrhea	4 (11.1%)	5 (5.5%)	.266
*Laboratory findings at diagnosis*			
WBC count (×10^3^/μL)	5.28 *±* 1.92	6.00 *±* 2.73	.406
Leukocytosis	3 (8.3%)	2 (2.2%)	.138
Leukopenia	9 (25.0%)	24 (26.4%)	>.999
Lymphopenia	9 (25.0%)	27 (29.3%)	.511
CRP (mg/dL)	1.4 *±* 3.6	3.2 *±* 4.9	< .001

Note: DM, diabetes mellitus; HTN, hypertension; COPD, chronic obstructive pulmonary disease; CRP, C-reactive protein.

The mean interval between diagnosis and admission in the CT-positive group was longer than that in the CT-negative group; however, the difference was not significant (1.3 ± 2.7 days vs. 0.8 ± 3.3 days; *P* = .142). The mean interval between symptom onset and diagnosis in the CT-negative group was insignificantly longer than that in the CT-positive group (3.8 ± 4.4 days vs. 3.2 ± 3.3 days; *P* = .433). At the time of diagnosis, fever and dyspnea were significantly more frequent in the CT-positive group than in the CT-negative group (39.6% vs. 19.4% and 13.2% vs. 0%, respectively; all *P* < .05). During the follow-up period, fever and dyspnea were again significantly more common in the CT-positive group than in the CT-negative group (62.6% vs. 33.3% and 30.4% vs. 8.3%, respectively; all *P* < .05). In the CT-positive group, 12 patients (13.2%) and 4 patients (4.4%) did not present any symptoms at the time of diagnosis and during the follow-up period, respectively.

Of the laboratory findings, WBC counts and the presence of leukocytosis and leukopenia did not significantly differ between the groups. However, the CRP level at diagnosis was significantly higher in the CT-positive group than in the CT-negative group (1.4 ± 3.6 mg/dL vs. 3.2 ± 4.9 mg/dL; *P* < .001).

The mean interval between diagnosis and chest CT was longer in the CT-positive group than in the CT-negative group; however, the difference did not achieve statistical significance (3.6 ± 4.4 days vs. 2.4 ± 2.8 days; *P* = .094). S1 Table lists the detailed CT findings in the CT-positive group.

### Differences in viral load between the groups

Table 2 summarizes the comparison of viral load between the CT-negative and CT-positive groups. Ct values were obtained within 1 day of the chest CT date, and the interval between chest CT and Ct value attainment was not significantly different between groups (0.7 ± 0.6 days vs. 0.7 ± 0.8 days, *P* = .994). The Ct value from the lower respiratory tract of CT-positive patients was significantly lower than that from CT-negative patients (26.7 ± 7.0 vs. 30.9 ± 8.4; *P* = .017), whereas the Ct values from the upper respiratory tract did not differ significantly between both groups (28.0 ± 6.8 vs. 27.2 ± 8.2; *P* = .566).

**Table 2:** Differences in viral load between groups.

	CT negative	CT positive	*P* value
Ct value from the upper respiratory tract	27.2 *±* 8.2	28.0 *±* 6.8	.566
Ct value from the lower respiratory tract	30.9 *±* 8.4	26.7 *±* 7.0	.017

**Fig 2:**
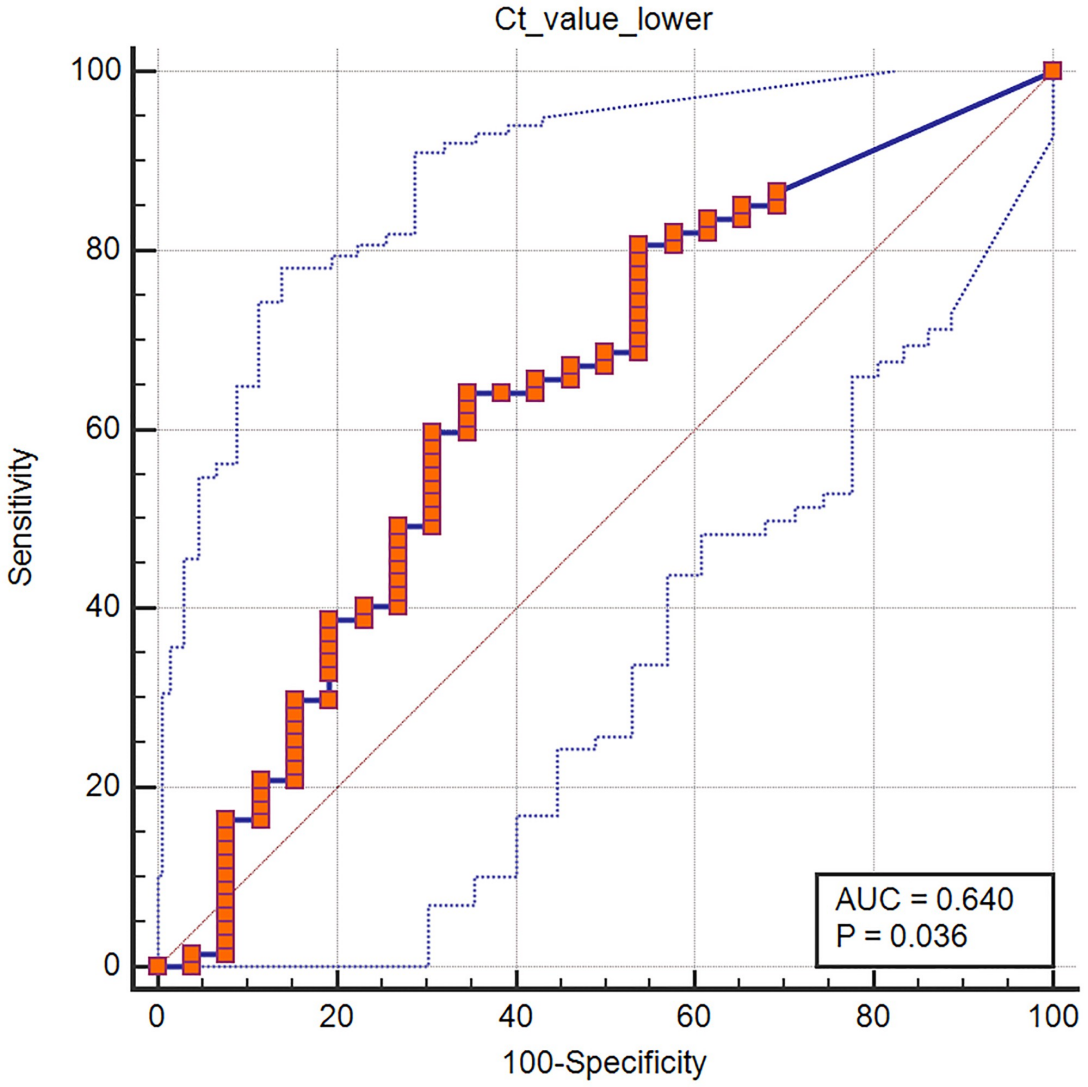
Receiver operating characteristic (ROC) curve showing the diagnostic performance of the lowest Ct value from the lower respiratory tract for predicting pneumonia on the initial chest CT. The AUC of the ROC curve of the lowest Ct value from the lower respiratory tract for predicting the presence of initial pneumonia was 0.640 (95% confidence interval [CI], 0.534–0.737; *P* = 0.036).

The AUC of the ROC curve of the lowest Ct value from the lower respiratory tract for predicting the presence of initial pneumonia was 0.640 (95% confidence interval [CI], 0.534–0.737; *P* = .036; Fig 2). The threshold (lowest) Ct value for the lower respiratory tract for predicting the presence of pneumonia on the initial chest CT was 27.71 (sensitivity and specificity of 64.2% and 65.4%, respectively).

### Clinical outcomes

The comparisons of clinical outcomes of both groups are summarized in Table 3. The mean length of hospital stay (days from first symptom to discharge) was significantly shorter in the CT-negative group than in the CT-positive group (17.3 ± 7.4 days vs. 22.2 ± 8.5 days; *P* = 0.014). No patient in the CT-negative group required ICU admission, whereas 12 (13.2%) patients required ICU admission in the CT-positive group (*P* = .018). Two (5.6%) and 5 (5.4%) patients in the CT-negative and CT-positive groups, respectively, died (*P* >.999).

**Table 3:** Comparison of clinical outcomes of both groups.

	CT negative	CT positive	*P* value
*Clinical outcomes*			
Discharged	22 (61.1%)	54 (58.7%)	.844
Duration of hospitalization^a^ (days between first symptom and discharge)	17.3 *±* 7.4	22.2 *±* 8.5	.014
ICU care	0	12 (13.2%)	.018
Duration of ICU care ^b^	0	17.8 *±* 12.5	N/A
Death	2 (5.6%)	5 (5.4%)	>.999
Days between diagnosis and death date	28.5 *±* 16.1	30.1 *±* 16.9	.756
*O*_*2*_ *therapy*			
Any of O_2_ therapy	6 (16.7%)	39 (42.4%)	.007
Nasal prongs	6 (16.7%)	34 (37.0%)	.033
Facial mask	1 (2.8%)	8 (8.7%)	.443
High-flow nasal prongs	2 (5.6%)	11 (12.0%)	.349
MV	1 (2.8%)	12 (13.0%)	.109
ECMO	0	4 (4.3%)	.576
*Severity classification according to the WHO*			
Critical COVID-19	1 (2.8%)	12 (13.0%)	.020
Severe COVID-19	5 (13.9%)	27 (29.3%)	
Non-severe COVID-19	30 (83.3%)	53 (57.6%)	

Note: ICU, intensive care unit; MV, mechanical ventilation; ECMO, extracorporeal membrane oxygenation.

^a^Patients in the hospital at the time of data collection were included.

^b^Patients under ICU care were included at the time of data collection.

A significantly greater number of patients required O_2_ therapy in the CT-positive group than in the CT-negative group (42.4% vs. 16.7%; *P* = .007). All O_2_ therapy modalities, including nasal prongs, facial masks, high-flow nasal prongs, MV, and ECMO, were more frequently used in the CT-positive group than in the CT-negative group, and nasal prongs were used significantly more frequently in the CT-positive group than in the CT-negative group (37.0% vs. 16.7%; *P* = .033). Deaths occurred while the patients were on O_2_ therapy in both groups.

The incidence of both clinically severe and critical COVID-19 infections were higher in the CT-positive group than in the CT-negative group (all *P* < .05).

### Factors present at the time of diagnosis associated with positivity on initial CT

The results of the univariate and multivariate analyses to identify factors present at the time of diagnosis associated with COVID-19 pneumonia on the initial chest CT are listed in Table 4. In the univariate analysis, age (odds ratio [OR], 1.049; CI, 1.024–1.073; *P* < .001), hypertension (hazard ratio [HR], 2.814; CI, 1.159–6.830; *P* = .022), fever at diagnosis (OR, 2.712; CI, 1.074–6.847; *P* = .035), and Ct value of the lower respiratory tract sample (OR, 0.928; CI, 0.873–0.987; *P* = .017) were associated with initial pneumonia. Multivariate logistic regression analysis was performed for the variables that were significantly associated with CT positivity (*P* < 0.05) in the univariate analysis. Among those factors, only age (OR, 1.060; CI, 1.020–1.102; *P* = .003) was significantly associated with initial COVID-19 pneumonia.

**Table 4:** Univariate and multivariate analyses of factors present at the time of diagnosis associated with positivity on initial CT.

	Univariate analysis			Multivariate analysis		
Factors	OR	95% CI	*P* value	OR	95% CI	*P* value
Age	1.049	1.024–1.073	< .001	1.060	1.020–1.102	.003
HTN	2.814	1.159–6.830	.022			
COPD	0.778	0.068–8.852	.839			
Cancer	2.882	0.342–24.302	.330			
Fever at diagnosis	2.712	1.074–6.847	.035			
Cough at diagnosis	0.723	0.328–1.597	.423			
Sputum at diagnosis	0.924	0.363–2.351	.868			
Ct value in the upper respiratory tract	1.016	0.952–1.084	.630			
Ct value in the lower respiratory tract	0.928	0.873–0.987	.017			
CRP	1.133	0.990–1.297	.070			

Note: OR, odds ratio; CI, confidence interval; HTN, hypertension; COPD, chronic obstructive pulmonary disease; CRP, C-reactive protein.

### Factors present at the time of diagnosis associated with O_2_ therapy

The results of the univariate and multivariate analyses to identify factors present at the time of diagnosis associated with the need for O_2_ therapy are listed in Table 5. In the univariate analysis, age (OR, 1.067; CI, 1.038–1.098; *P* < .001), hypertension (OR, 3.570; CI, 1.666–7.650; *P* = .001), cancer (OR, 15.105; CI, 1.795–127.147; *P* = .012), fever at diagnosis (OR, 3.450; CI, 1.587–7.500; *P* = .002), dyspnea at diagnosis (OR, 29.625; CI, 9.211–95.279; *P* < 0.001), lymphopenia (OR, 3.824; CI, 1.667–8.771; *P* = .002), CRP level (OR, 1.448; CI, 1.219–1.719; *P* < .001), and positivity on initial CT (OR, 3.679; CI, 1.396–9.697; *P* = .008) were associated with the need for O_2_ therapy. Multivariate logistic regression analysis was performed for the variables that were significantly associated with CT positivity (*P* < .05) in the univariate analysis. Among those factors, age (OR, 1.062; CI, 1.014–1.112; *P* = .011), fever at diagnosis (OR, 6.689; CI, 1.715–26.096; *P* = .006), and CRP level (OR, 1.393; CI, 1.150–1.687; *P* = .001) were significantly associated with the need for O_2_ therapy.

**Table 5:** Univariate and multivariate analyses of factors at the point of diagnosis for the need of O_2_ therapy.

	Univariate analysis			Multivariate analysis		
Factors	OR	95% CI	*P* value	OR	95% CI	*P* value
Age	1.067	1.038–1.098	< .001	1.062	1.014–1.112	.011
HTN	3.570	1.666–7.650	.001			
COPD	3.814	0.336–43.264	.280			
Cancer	15.105	1.795–127.147	.012			
Smoking	2.706	0.926–7.905	.069			
Fever at diagnosis	3.450	1.587–7.500	.002	6.689	1.715–26.096	.006
Cough at diagnosis	1.827	0.860–3.880	.117			
Sputum at diagnosis	2.067	0.870–4.912	.100			
Dyspnea at diagnosis	29.625	9.211–95.279	< .001			
Ct value in the upper respiratory tract	0.690	0.942–1.052	.648			
Ct value in the lower respiratory tract	0.913	0.926–1.038	.913			
Lymphopenia	3.824	1.667–8.771	.002			
CRP	1.448	1.219–1.719	< .001	1.393	1.150–1.687	.001
Initial CT positive	3.679	1.396–9.697	.008			

Note: OR, Odds ratio; CI, confidence interval; HTN, hypertension; COPD, chronic obstructive pulmonary disease; CRP, C-reactive protein.

### Follow-up data from the CT-negative group

Among 36 patients in the CT-negative group, 3 patients (8.3%) developed COVID-19 pneumonia, as confirmed with follow-up chest CT (n = 1) and CXRs (n = 2), and the pneumonia developed within 5.3 ± 1.5 days from the initial chest CT.

In the CT-negative group, the patients who developed pneumonia were significantly older those who did not (40.3 ± 21.1 vs. 72.0 ± 10.8; *P* = .023). With regard to the laboratory findings, the CRP level was significantly higher in patients who developed pneumonia than in those who did not (1.0 ± 2.7 vs. 5.4 ± 8.7; *P* = .031). In addition, the Ct value from the lower respiratory tract samples of patients who developed pneumonia was significantly lower than that of patients who did not (18.1 ± 5.9 vs. 31.9 ± 7.7, *P* = .034); the Ct value from the upper respiratory tract of patients who developed pneumonia was lower than that of patients who did not; however, the difference was not statistically significant (18.2 ± 0.9 vs. 28.1 ± 8.0; *P* = .065).

## Discussion

This study presents the differences in the clinical findings, viral load, and outcomes between patients with confirmed COVID-19 who initially presented normal chest CT findings compared with those who initially had positive CT findings. To our knowledge, this report is the largest multicenter case series data of hospitalized patients with COVID-19 who initially presented normal chest CT findings.

Our study established an association between viral load and chest CT findings, and several studies have shown that the viral load is associated with severity of COVID-19 infection. Liu et al. suggested that higher viral loads might be associated with severe clinical outcomes because the mean viral load in severe cases was 60 times higher than that in mild cases [13]. Chen et al. reported that viral load is associated with elevated interleukin-6 levels, which is likely a part of a larger cytokine storm, and with poor prognosis in patients with COVID-19 [14]. Our results showed a significantly higher viral load from lower respiratory tract samples in CT-positive patients than in CT-negative patients. Furthermore, among the initially CT-negative patients, those who later developed pneumonia had a significantly higher viral load than those who did not. In addition, per our results, the threshold Ct value for the lower respiratory tract for predicting COVID-19 pneumonia using chest CT was 27.71. Considering that the Ct value from the lower respiratory tract sample was associated with the presence or absence of pneumonia, the Ct threshold value has predictive utility for the development of COVID-19 pneumonia.

Older age is a prominent risk factor for severe COVID-19 outcomes and COVID-19-related death [15,16]. In our study, patients who were initially CT positive were significantly older than those who were initially CT negative. The multivariate analysis showed that age was the only factor that could predict pneumonia in patients with initially positive CT findings and the factor associated with the need for O_2_ therapy. In addition, among the 36 initially CT-negative patients, the 3 (8.3%) who developed COVID-19 pneumonia later were significantly older than those who did not. Furthermore, the proportion of patients with hypertension was higher in the CT-positive group than in the CT-negative group. In addition, fever at diagnosis and CRP level were factors associated with the need for O_2_ therapy. According to a previous study, hypertension is a risk factor for COVID-19-related fatality, and fever at admission is a risk factor for O_2_ therapy [17,18]. Therefore, the results of our study also reveal the importance of these risk factors with regard to COVID-19 outcomes.

Our study also described the clinical symptoms, clinical course, and laboratory findings of patients who were initially CT negative. Moreover, 12 CT-negative patients (13.2%) were asymptomatic, and 33 initially CT-negative patients (91.7%) presented with respiratory symptoms. Although the incidence of fever or dyspnea at diagnosis was higher among CT-positive patients, determining the presence or absence of COVID-19 pneumonia based only on symptomatology is challenging. In addition, because a significant percentage of the CT scans were negative in our study, the initial radiological examination may not be sufficient for screening for or discriminating COVID-19. In our study, 8.3% of the initially CT-negative patients later developed COVID-19 pneumonia. These patients were significantly older, had significantly higher initial CRP levels, and had a significantly higher viral load in the lower respiratory tract than those CT-negative patients who did not develop pneumonia. We cannot suggest a threshold viral load value because too few patients with pneumonia were included. However, the possibility of pneumonia should be considered in patients with advanced age, high CRP levels, and high viral loads even if they have negative initial chest CT findings.

In our study, the initially CT-positive patients had worse clinical outcomes and longer hospital stays than the CT-negative patients; however, the number of discharged patients in both groups did not differ significantly. No initially CT-negative patient required ICU admission in our study. Furthermore, the number of patients who required O_2_ therapy was significantly greater among the initially CT-positive patients. In addition, initial CT positivity was a significant factor associated with the need for O_2_ therapy in the univariate analysis (OR, 3.679; CI, 1.396–9.697; P = .008). Although initial CT positivity had no statistical significance in the multivariate analysis, this may be because other factors were more strongly associated with outcome. Nevertheless, initial CT positive is a significant factor in univariate analysis and can be considered as a factor that is clinically related to outcome. Although chest CT may have limited utility in initial screening and diagnosis of COVID-19, our results show that the initial chest CT findings have prognostic utility.

It may be difficult to apply our results to the use of initial CXRs, which have lower lesion detectability than chest CT (i.e., even if CXRs show negative findings, there may be lesions on CT). However, a previous study also showed significant differences in viral load and recovery times between patients with and without pneumonia proven by CXRs in young asymptomatic and mildly symptomatic patients [19]. Therefore, we suggest that patients with initial pneumonia detected with any imaging modality should be treated more carefully than patients with negative findings. In addition, several clinical factors present at the time of diagnosis associated with positivity on initial CT or future O_2_ therapy were identified in this study. Therefore, even if performing a chest CT scan is difficult, it will be helpful to manage patients if a management plan is made according to these risk factors. This study reflects the naïve course of COVID-19 infection because it was conducted in a study cohort from early in the pandemic when symptomatic treatment was mostly provided. In the case of an initial CT positive, if the currently recommended treatments (i.e., steroid treatment) are applied earlier, an improved clinical course and prognosis may be shown. Therefore, the study design should be planned considering this point in future studies.

This study has several limitations. First, it was retrospective in nature, and we only selected patients who underwent initial chest CT, which introduced selection bias. However, our study is valuable because it was a multicenter study that included patients from 9 hospitals, making for the largest population of patients with COVID-19 who were initially CT-negative. We presented the clinical outcomes and viral load data of these patients and discussed the pertinent literature. Second, because of our country’s patient privacy protection policies, we could not review all the CT images, and only the CT reports from board-certified thoracic radiologists at each hospital were provided in most cases. However, because normal chest CT findings were clearly distinguishable from the reports, the overall analysis remains reliable. Third, during the early days of the COVID-19 pandemic, hospitalization was continued until 2 consecutive negative rRT-PCR results in our country. Therefore, the definition of hospitalization likely differs from that used in studies from other countries. In addition, state-designated isolation beds were managed similarly to the ICU with the use of MV or ECMO allowed. As such, some patients who received MV or ECMO were counted as not requiring ICU admission.

In conclusion, initially CT-negative patients had better outcomes than initially CT-positive patients. Age was the only factor associated with initially positive CT findings. The viral load in initially CT-positive patients was significantly higher than that in initially CT-negative patients. We report the threshold Ct value of 27.71 to predict COVID-19 pneumonia in initially CT-positive patients.

## Supporting information

S1 Table Detailed CT findings in the CT-positive group. This table lists the detailed CT findings in the CT-positive group.
(DOCX)

S1 Dataset (XLSX)
