# Supplementary material for: Clinical findings, viral load, and outcomes of COVID-19: Comparison of patients with negative and positive initial chest computed tomography
Source: PLoS One. 2022 Mar 3;17(3):e0264711. doi: 10.1371/journal.pone.0264711 (PMC8893619; doi:10.1371/journal.pone.0264711)
Supplement: S1 Table — This table lists the detailed CT findings in the CT-positive group. (DOCX) [file pone.0264711.s001.docx]

**S1 Table. Detailed CT findings in the CT-positive group.** This table lists the detailed CT findings in the CT-positive group.

| *Pattern* |  |
| --- | --- |
| Only GGO | 45 (49.5%) |
| Both GGO and consolidation | 40 (44.0%) |
| Only consolidation | 7 (7.6%) |
| *No of affected lobes* |  |
| 1 | 16 (17.4%) |
| 2 | 20 (21.7%) |
| 3 | 10 (10.9%) |
| 4 | 2 (2.2%) |
| 5 | 43 (46.7%) |
| *More than two lobes affected* | 58 (63.0%) |
| *Bilateral lung disease* | 72 (78.3%) |
| *Affected lobes* |  |
| Right upper lobe | 57 (62.0%) |
| Right middle lobe | 46 (50.0%) |
| Right lower lobe | 74 (80.4%) |
| Left upper lobe | 58 (63.0%) |
| Left lower lobe | 75 (81.5%) |
| *Opacification distribution* |  |
| Rounded morphology | 11 (12.0%) |
| Linear opacities | 3 (3.3%) |
| Crazy-paving pattern | 3 (3.3%) |
| Peripheral/peribronchovascular distribution | 69 (75.0%) |
| COP pattern | 3 (3.3%) |
| *Other findings* |  |
| None | 76 (82.6%) |
| Discrete pulmonary nodules | 2 (2.2%) |
| Pleural effusion | 5 (5.4%) |
| Lymphadenopathy | 4 (4.3%) |
| Pulmonary emphysema | 4 (4.3%) |
| Pulmonary fibrosis | 1 (1.1%) |
